# Isoferulic Acid, a New Anti-Glycation Agent, Inhibits Fructose- and Glucose-Mediated Protein Glycation *in Vitro*

**DOI:** 10.3390/molecules18066439

**Published:** 2013-05-30

**Authors:** Aramsri Meeprom, Weerachat Sompong, Catherine B. Chan, Sirichai Adisakwattana

**Affiliations:** 1 Program in Clinical Biochemistry and Molecular Medicine, Department of Clinical Chemistry, Faculty of Allied Health Sciences, Chulalongkorn University, Bangkok 10330, Thailand; E-Mails: Jamaea_p@hotmail.com (A.M.); Kradat_pup@hotmail.com (W.S.); 2 Research Group of Herbal Medicine for Prevention and Therapeutic of Metabolic Diseases, Chulalongkorn University, Bangkok 10330, Thailand; 3 Department of Physiology and Department of Agricultural, Food and Nutritional Sciences, University of Alberta, Edmonton, AB T6G 2R3, Canada; E-Mail: Cbchan@ualberta.ca; 4 Department of Nutrition and Dietetics, Faculty of Allied Health Sciences, Chulalongkorn University, Bangkok 10330, Thailand; E-Mail: Sirichai.a@chula.ac.th

**Keywords:** isoferulic acid, protein glycation, advanced glycation end products, fructosamine, protein oxidation, N^ε^-(carboxymethyl)lysine

## Abstract

The inhibitory activity of isoferulic acid (IFA) on fructose- and glucose-mediated protein glycation and oxidation of bovine serum albumin (BSA) was investigated. Our data showed that IFA (1.25–5 mM) inhibited the formation of fluorescent advanced glycation end products (AGEs) and non-fluorescent AGE [N^ε^-(carboxymethyl) lysine: CML], as well as the level of fructosamine. IFA also prevented protein oxidation of BSA indicated by decreasing protein carbonyl formation and protein thiol modification. Furthermore, IFA suppressed the formation of β-cross amyloid structures of BSA. Therefore, IFA might be a new promising anti-glycation agent for the prevention of diabetic complications *via* inhibition of AGEs formation and oxidation-dependent protein damage.

## 1. Introduction

Protein glycation is a non-enzymatic reaction between the carbonyl group of a reducing sugar and the free amino group of a protein. The reaction initiates a complex cascade of repeated condensations, rearrangements, and oxidative modifications, resulting in the reversible formation of a characteristic structure called a Schiff’s base [1,2]. This is further rearranged into more stable structures called Amadori products, which undergo further oxidation, generating dicarbonyl compounds to form cross-linked structures termed advanced glycation end products or AGEs [3,4]. One of the major chemical AGE structures is N**^ε^**-(carboxymethyl) lysine (CML) formed by oxidative cleavage of Amadori products and by generation of reactive α-oxoaldehydes from glucose [5,6]. AGEs play a vital role in further cross-linking or modification of other proteins and generating oxidizing intermediates, resulting in induction of oxidative stress in vascular cells as well as other tissues [7]. For this reason, the excessive formation of AGEs and their accumulation in the tissues is a significant contributor to age-related diseases [8] and diabetic complications such as retinopathy, nephropathy and neuropathy [9]. Current scientific evidence demonstrates that inhibition of AGEs formation is a therapeutic strategy for diabetic complications [10]. Aminoguanidine, a well-known anti-glycation agent, has received the most interest from a clinical trials perspective, and inhibits AGEs formation both *in vitro* and *in vivo* [9,11,12]. However, recent studies have indicated that aminoguanidine may have some toxicity when administered for diabetic nephropathy [13]. Therefore, much effort has been extended in search of phytochemical compounds from dietary plants, fruits, and herbal medicines that effectively inhibit AGE formation [3,14,15].

**Figure 1:**
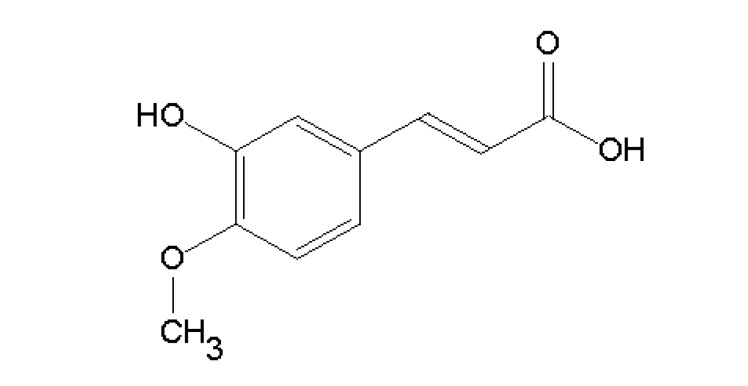
The chemical structure of isoferulic acid (3-hydroxy-4-methoxycinnamic acid).

*Cimicifuga heracleifolia* is a herbal medicine commonly found in oriental countries such as Japan and China that has been traditionally used as an anti-inflammatory drug [16,17]. The pharmacological properties of *C. heracleifolia* are attributable to a major active ingredient called isoferulic acid (Figure 1) which has been previously reported to have anti-inflammatory [17,18,19], anti-viral [20], anti-oxidative [21,22], and anti-diabetic properties [23]. Interestingly, the administration of isoferulic acid (IFA) reduces plasma glucose concentration in streptozotocin-induced diabetic rats by activating α_1_-adrenoceptors to enhance the secretion of β-endorphin, which can stimulate the opioid μ-receptors to increase glucose use or/and reduce hepatic gluconeogenesis [24]. However, the potential inhibitory effects of IFA on protein glycation have never been reported. Hence, we were particularly interested in investigating the effects of IFA on fructose- and glucose-mediated protein glycation. In addition, the study also examined the effects of IFA on oxidation-dependent damage to BSA and formation of CML *in vitro*. 

## 2. Results and Discussion

**Figure 2:**
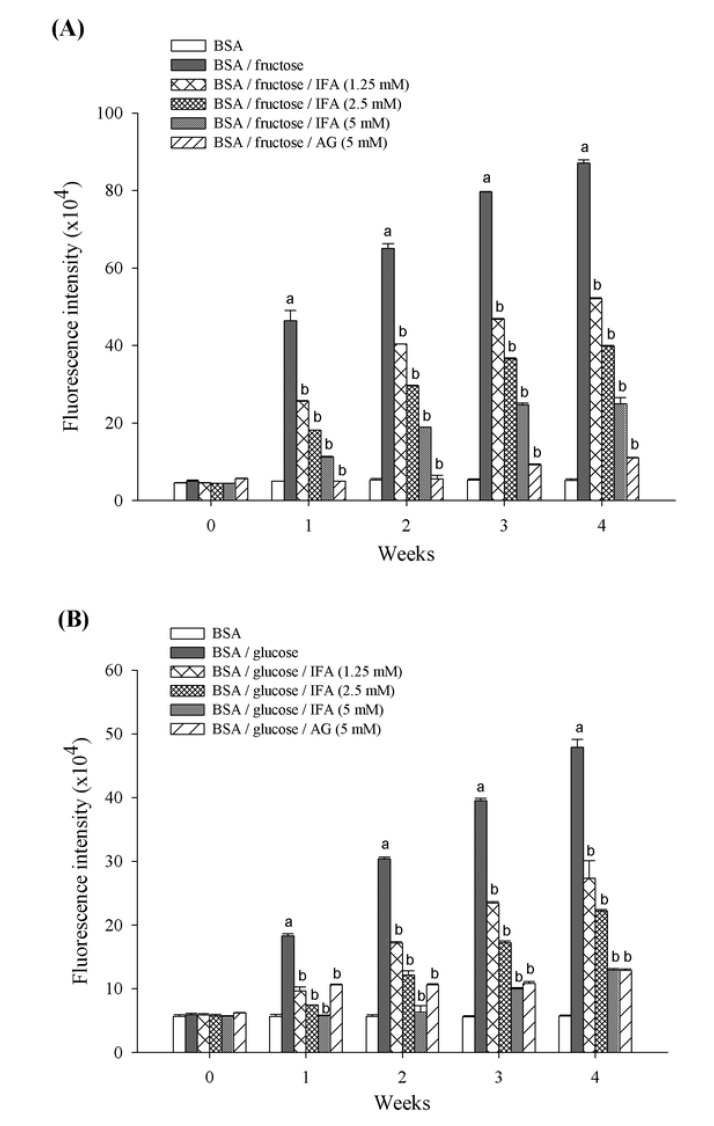
The inhibitory effect of IFA on the formation of fluorescent AGEs in BSA/fructose (**A**) and BSA/glucose (**B**) systems. Results are expressed as mean ± SEM (n = 3). ^a^ *p* < 0.05 when compared to BSA, ^b^ *p* < 0.05 when compared to BSA/fructose or glucose.

Protein glycation is a spontaneous chemical modification of proteins or amino acids by reducing monosaccharides such as glucose and fructose [15,25]. This reaction generates irreversible heterogeneous byproducts termed advanced glycation end products (AGEs) which are implicated in the development of aging as well as the pathogenesis of age-related disorders including Alzheimer’s disease and diabetes complications [26,27]. This is the first study to report the inhibitory effect of isoferulic acid (IFA) on fructose- and glucose-mediated BSA glycation. Our findings also indicate that the nonenzymatic fructation of albumin occurred at a much higher rate than did the glucation reaction, as reported by others [28,29]. The formation of AGEs was monitored weekly by measuring fluorescence intensity of the BSA-sugar solutions. As shown in Figure 2, as the duration of incubation increased there was significantly more BSA-glycation with both glucose and fructose. When IFA was added to reaction media containing BSA/fructose and BSA/glucose systems, a significant concentration-dependent reduction in the fluorescence intensity was noted throughout the study period. At week 4 of incubation, the percentage inhibition of AGEs formation by IFA at the concentrations of 1.25, 2.5, and 5 mM was 40.2%, 54.3%, and 71.4%, respectively for the BSA/fructose system, and 42.9%, 53.6%, and 73.0%, respectively, for the BSA/glucose system. In addition, aminoguanidine (AG) inhibited the formation of AGEs in BSA/fructose and BSA/glucose by 87.3% and 73.1%, respectively. 

**Figure 3:**
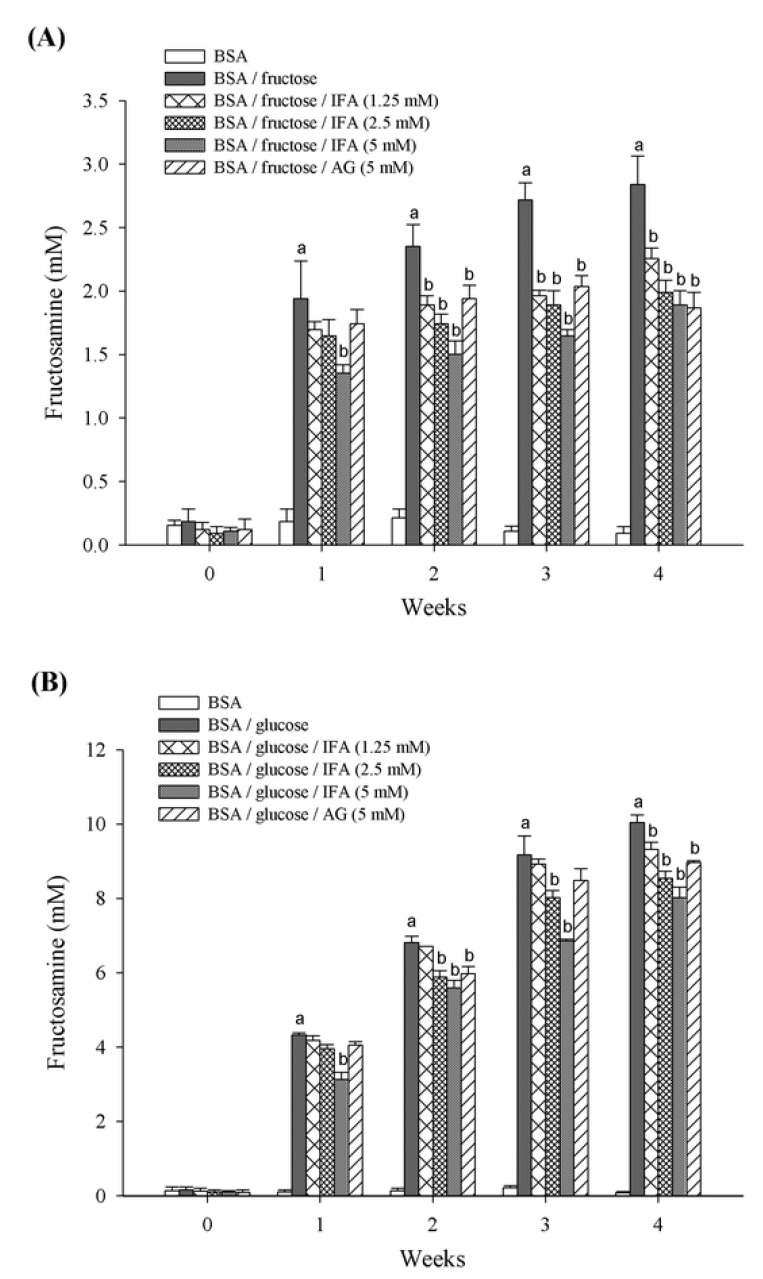
The effect of IFA on the Amadori production in BSA/fructose (**A**) and BSA/glucose (**B**) systems. Results are expressed as mean ± SEM (n = 3). ^a^ *p* < 0.05 when compared to BSA, ^b^ *p* < 0.05 when compared to BSA/fructose or glucose.

In the early stages of glycation, unstable Schiff’s bases are formed and turned into Amadori products such as fructosamine, which is clinically used as an indicator for short-term control of blood sugar in diabetic patients [15]. Reduction of fructosamine, therefore, is a therapeutic way to delay incident vascular complications [30]. We found that IFA markedly suppressed the formation of fructosamine as well as AGEs formation. The amount of Amadori product, fructosamine, is shown in Figure 3. Compared with non-glycated BSA, monosaccharide-induced glycated BSA was associated with significantly increased fructosamine after one week of study, however, less fructosamine was observed in the BSA/fructose than the BSA/glucose system. The addition of IFA and AG significantly suppressed the generation of fructosamine. At the end of the study period, concentrations of IFA of 1.25, 2.5, and 5 mM inhibited the formation of fructosamine in BSA/fructose by 20.6%, 30.0%, and 33.4%, and in BSA/glucose by 7.3%, 15.0%, and 20.1%, respectively, whereas the inhibitory effect of 5 mM AG was 34.3% and 10.7% in BSA/fructose and BSA/glucose, respectively. Thus, IFA was more effective in reducing fructosamine in the BSA/fructose system than the BSA/glucose system. 

**Figure 4:**
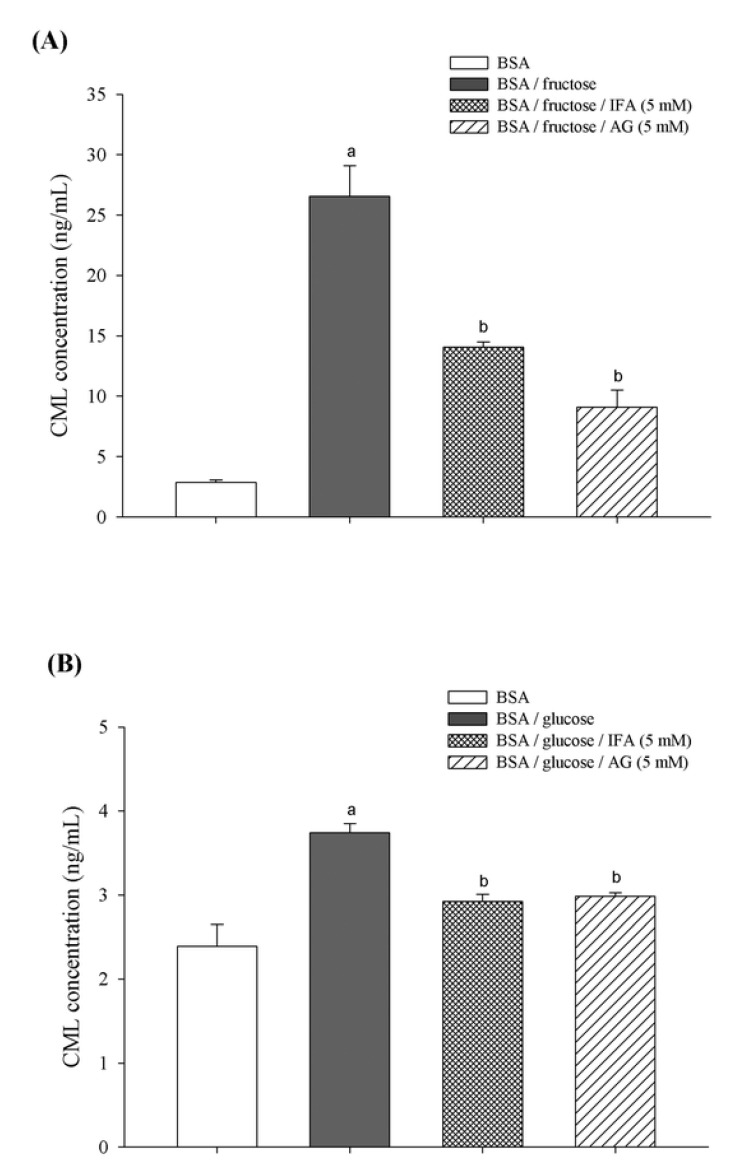
The effect of IFA on the formation of N^ε^-(carboxymethyl) lysine (CML) in BSA/fructose (**A**) and BSA/glucose (**B**) systems at week 4. Results are expressed as mean ± SEM (n = 3). ^a^ *p* < 0.05 when compared to BSA, ^b^ *p* < 0.055 when compared to BSA/fructose or glucose.

Furthermore, the production of N^ε^-(carboxymethyl)lysine (CML), an indicator of AGEs formation generated either from oxidative breakdown of Amodori product [31] or polyol pathway mediated by α-oxoaldehydes such as glyoxal, methylglyoxal, and 3-deoxyglucosone [32], was also inhibited by IFA both in fructose- and glucose-induced glycation. CML has been used as a biomarker for the formation of non-fluorescent AGE. Fructose-induced glycated BSA exhibited a 9.2-fold increase in CML formation [Figure 4(A)], whereas there was 1.6-fold increase in glycated BSA induced by glucose [Figure 4(B)] when compared to non-glycated BSA at week 4. The results showed that IFA at a concentration of 5 mM significantly inhibited the formation of CML by 47.0% in BSA/fructose and 21.9% in BSA/glucose. Likewise, AG significantly reduced the level of CML by about 65.8% and 20.2% for BSA/fructose and BSA/glucose systems, respectively.

**Figure 5:**
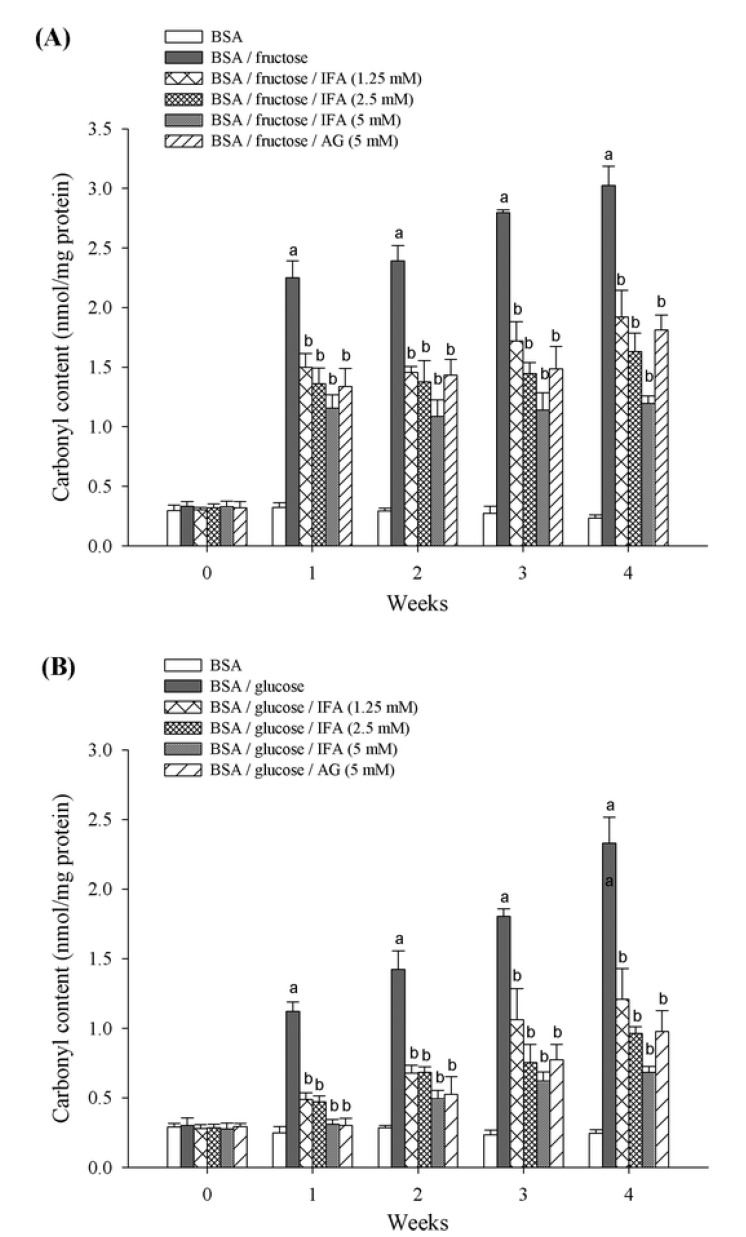
The effect of IFA on the protein carbonyl content in BSA/fructose and BSA/glucose systems. Results are expressed as mean ± SEM (n = 3). ^a^ *p* < 0.05 when compared to BSA, ^b^ *p* < 0.05 when compared to BSA/fructose or glucose.

Carbonyl content and thiol group formation was assessed as indicators of protein oxidation during the glycation process. As shown in Figure 5, the carbonyl content of glycated BSA in week 2 and 4 of the study period was significantly higher than non-glycated BSA. The magnitude of increase was approximately 8.2- and 12.9-fold in the BSA/fructose system, and 5.0- and 9.5-fold in the BSA/glucose system, respectively. At week 4 of incubation, IFA (1.25–5 mM) reduced the level of protein carbonyl by 36.5%, 46.1%, and 60.5% in BSA/fructose, and 48.1%, 58.7%, and 70.6% in BSA/glucose, respectively. In addition, AG decreased the protein carbonyl content by 40.2% and 58.1% in BSA/fructose and BSA/glucose, respectively.

**Figure 6:**
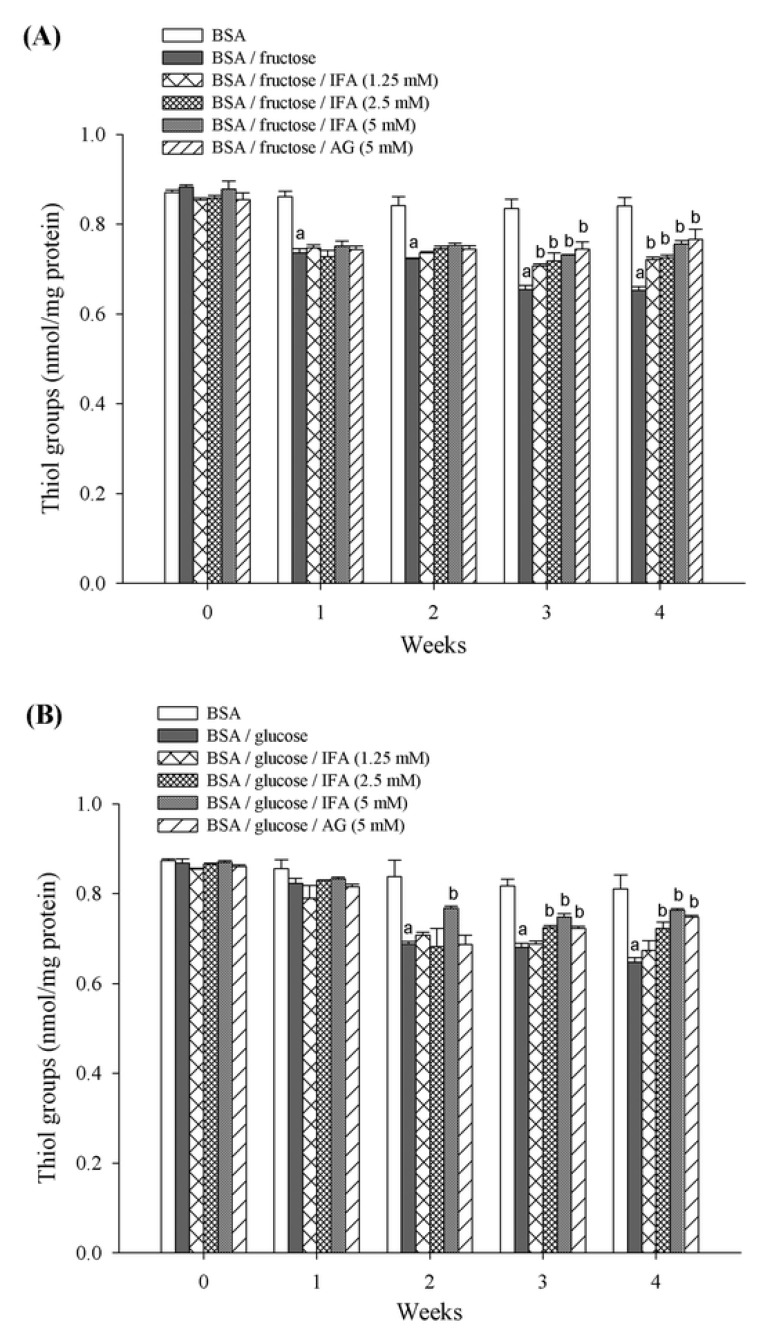
The effect of IFA on protein thiol formation in BSA/fructose and BSA/glucose systems. Results are expressed as mean ± SEM (n = 3). ^a^ *p* < 0.05 when compared to BSA, ^b^ *p* < 0.05 when compared to BSA/fructose or glucose.

The effects of IFA on the oxidation of protein thiols are shown in Figure 6. BSA incubated with fructose or glucose had significantly decreased thiol groups when compared to BSA alone. At week 2 and 4, fructose caused a decrease in thiol groups in BSA by 14.1% and 22.4%, respectively, whereas the loss of thiol group in BSA mediated by glucose was 17.9% and 20.2%, respectively. There was a significant improvement in the level of thiol after addition of IFA at various concentrations (1.25–5 mM) as well as AG. At week 4 of incubation, IFA- and AG-mediated prevention of loss thiol groups was 10.6%, 11.3%, 16.0%, and 17.5% for BSA/fructose, and 4.2%, 11.7%, 18.9%, and 15.6% for BSA/glucose, respectively.

Glycation is not only a major cause of AGE-mediated protein modification, but it also induces oxidation-dependent tissue damage, leading to development of complications of diseases including diabetes [33,34]. An increase in carbonyl content and the loss of free thiol groups by modification of cysteine residues is a direct reflection of protein oxidation of BSA [35,36]. Reactive oxygen species generated during glycation and glyoxidation, such as superoxide, are able to oxidize side chains of amino acid residues in protein to form carbonyl derivatives and also diminish an oxidative defense of protein by decreasing thiol groups, leading to damage of cellular proteins [37]. Apart from a significant role in directly resisting against these free radicals, most antioxidants also possess the ability to inhibit oxidative damage-mediated glycation and glycoxidation by modulating abnormal levels of protein carbonyls and thiol groups [15,37]. A marked increase in protein carbonyl formation and oxidation of thiols was observed when BSA reacted with fructose and glucose. In the present study, IFA significantly suppressed protein carbonyl formation and oxidation of thiols in BSA. Several previous studies demonstrated that IFA potently reduced oxidative stress and free radicals *in vitro*, including scavenging of DPPH and ABTS radicals [21,38], superoxide anion and hydroxyl radicals, as well as reduction of lipid peroxidation [22]. Thus, the ability of IFA to modulate glycation-mediated BSA oxidation might be from its anti-oxidant activity. However, other underlying mechanisms of anti-glycation may also be relevant. Metal chelating activity appeared to be one of important actions mediated anti-glycation property. Chelation, by inhibition of autoxidative glycosylation and glycoxidation, would delay the progression from AGE precursor to AGE cross-link. Consequently, this activity enables the rejuvenation of the extracellular matrix by turnover of cross-linked proteins and biosynthesis of native matrix proteins [39]. Furthermore, metal chelating agents could also inhibit enzymatic and metal-catalyzed ROS production after ligation of AGEs with scavenger receptors, such as RAGE. There has been the evidence regarding copper-ion chelating activity of AG [40]. Likewise, IFA has been found to be a metal ion chelating agent [22]. From this point, metal chelating activity of IFA might be one of possible mechanism responsible for inhibition of glycation. Recently, scientists have proposed other anti-glycation mechanisms including breaking the cross-linking structures in the formed AGEs, blocking the carbonyl or dicarbonyl groups in reducing sugars, Schiff’s bases or Amadori products, and inhibiting the formation of late-stage Amadori products [31,41]. Further comprehensive studies of IFA are required to evaluate the anti-glycation mechanisms described above.

**Figure 7:**
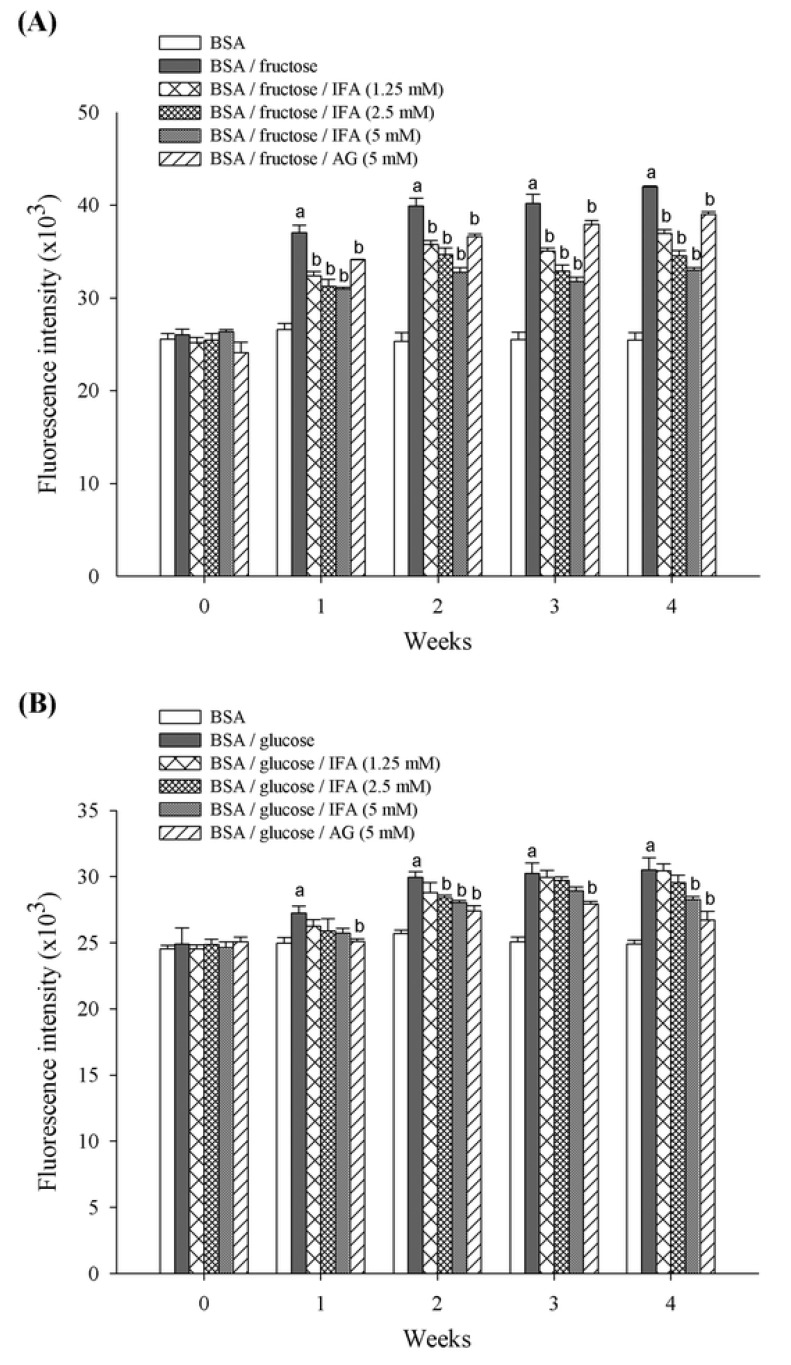
The effect of IFA on the amyloid cross β-structure of BSA/fructose (**A**) and BSA/glucose (**B**) systems. Results are expressed as mean ± SEM (n = 3). ^a^ *p* < 0.05 when compared to BSA, ^b^ *p* < 0.05 when compared to BSA/fructose or glucose.

The thioflavin T assay is commonly used to quantify the amount of a protein modification called amyloid cross-β structure in glycated BSA. As shown in Figure 7, compared with non-glycated BSA, the glycated BSA exhibited elevated amyloid cross-β conformation up to 1.6-fold (fructose) and 1.2-fold (glucose). IFA at concentrations of 1.25–5 mM reduced the level of amyloid cross-β structure in a concentration-dependent manner for both BSA/fructose (12.0%, 17.7%, and 21.5%) and BSA/glucose (0.2%, 3.1%, and 7.3%) systems. Similarly, a significant decrease in the level of amyloid cross-β structure (7.1% in BSA/fructose and 12.4% in BSA/glucose) was observed in the presence of AG at week 4 of incubation.

Protein glycation is an important factor affecting conformational changes of polypeptides, especially the amyloid cross-β structure [42]. Amyloid cross-β is an aggregated structure of protein that accumulates as deposited fibrils in the brains of patients with glycation-related diseases such an Alzheimer’s [8,43]. Moreover, β-amyloid deposits in the pancreas have also been found to be pathologic lesions in pancreatic β-cells of type 2 diabetic patients [44]. Notably, accumulation of protein aggregation induces pancreatic islet amyloidosis, which directly damages β-cell and impairs insulin secretion [45,46]. Our findings show that the formation of amyloid cross-β structure can be reduced by IFA. This beneficial effect of IFA might help to reduce the risk of developing debilitating degenerative diseases in diabetic patients. 

## 3. Experimental

### 3.1. Chemicals and Reagents

Isoferulic acid (3-hydroxy-4-methoxycinnamic acid), 5,5'-dithiobis(2-nitrobenzoic acid) (DTNB), thioflavin T, nitroblue tetrazolium (NBT), 1-deoxy-1-morpholino-D-fructose (DMF), L-cysteine, and aminoguanidine were obtained from Sigma (St. Louis, MO, USA). 2,4-dinitrophenylhydrazine (DNPH) was purchased from Ajax Finechem (Taren Point, Australia). Trichloroacetic acid (TCA) and guanidine hydrochloride were purchased from Merck (Darmstadt, F.R., Germany) and Fluka (Steinheim, Germany), respectively. OxiSelect™ N^ε^-(carboxymethyl) lysine (CML) ELISA kit was purchased from Cell Biolabs (San Diego, CA, USA). All other chemicals used were of analytical grade.

### 3.2. *In Vitro* Glycation of Bovine Serum Albumin (BSA)

The formation of glycated BSA formation was done according to a previous method with minor modifications [4]. BSA (10 mg/mL) was incubated with glucose or fructose (0.5 M) in 0.1 M phosphate buffer (PBS), pH 7.4 containing 0.02% sodium azide (NaN_3_) at 37 °C for 4 weeks in the absence or presence of IFA (1.25, 2.5, and 5 mM) and aminoguanidine (5 mM). Dimethylsulfoxide (DMSO, 4%) was used as a solvent for this study. Aliquots of the reaction mixtures were then assayed for AGEs formation, fructosamine, protein carbonyl content, thiol group, amyloid cross β structure, and CML.

### 3.3. Determination of AGEs Formation

The formation of AGEs was determined spectrofluorometrically (Wallac 1420 Victor^3^ V, PerkinElmer, Walham, MA, USA) at excitation and emission wavelengths of 355 and 460 nm, respectively. The inhibitory effect of IFA and aminoguanidine was evaluated by the calculation of percentage inhibition compared with maximum glycation elicited by glucose or fructose.

### 3.4. Fructosamine Measurement

The concentration of the Amadori product fructosamine was measured by NBT assay [15]. Briefly, glycated BSA (10 μL) was incubated with 100 μL of 0.5 mM NBT in 0.1 M carbonate buffer, pH 10.4 at 37 °C. The absorbance was measured at 530 nm at 10 and 15 min time points. The concentration of fructosamine was calculated compared to DMF as the standard.

### 3.5. Determination of N^ε^-(carboxymethyl) Lysine (CML)

N^ε^-(carboxymethyl) lysine (CML), a major antigenic AGE structure, was determined using an enzyme linked immunosorbant assay (ELISA) kit according to the manufacturer’s protocol. The absorbance of samples was compared with CML-BSA standard provided in the assay kit.

### 3.6. Determination of Protein Carbonyl Content

The carbonyl group in glycated BSA, a marker for protein oxidative damage, was assayed according to the method of Levine and colleagues with minor modifications [47]. Briefly, 400 µL of 10 mM DNPH in 2.5 M HCl was added to 100 µL of glycated samples. After 1 h incubation in the dark, 500 µL of 20% (w/v) TCA was used for protein precipitation (5 min on ice) and then centrifuged at 10,000 *g* for 10 min at 4 °C. The protein pellet was washed with 500 µL of ethanol/ethyl acetate (1:1) mixture three times and resuspended in 250 µL of 6 M guanidine hydrochloride. The absorbance was measured at 370 nm. The carbonyl content of each sample was calculated based on the extinction coefficient for DNPH (ε = 22,000 M^−1^cm^−1^). The results were expressed as nmol carbonyl/mg protein.

### 3.7. Thiol Group Estimation

The free thiols in glycated samples were measured by Ellman’s assay with minor modifications [48]. Briefly, 70 μL of glycated samples were incubated with 5 mM DTNB in 0.1 M PBS, pH 7.4 at 25 °C for 15 min. The absorbance of samples was measured at 410 nm. The concentration of free thiols was calculated from L-cysteine standard and expressed as nmol/mg protein.

### 3.8. Thioflavin T Assay

Thioflavin T, a marker of amyloid cross-β structure, was measured according to a previous method with minor modifications [49]. Briefly, 100 μL of 64 µM thioflavin T in 0.1 M PBS, pH 7.4 was added to the glycated samples (10 μL) and incubated at 25 °C for 60 min. The fluorescence intensity was measured at excitation wavelength of 435 nm and emission wavelength of 485 nm.

### 3.9. Statistical Analysis

All data are presented as means ± SEM. Statistical significance was evaluated using one-way ANOVA. A Duncan post-hoc comparison was used to analyze the sources of significant differences. A *p*-value < 0.05 was considered statistically significant.

## 4. Conclusions

Our findings demonstrate that IFA protects against fructose- and glucose-mediated glycation *in vitro*. Additionally, IFA reduces the level of fructosamine, the formation of CML, and amyloid cross-β structure. It also decreases the protein carbonyl content and prevents thiol group modification in BSA. The beneficial effects of IFA may be applied to prevention or management of AGE-mediated pathologies, particularly for those who are at risk of developing diabetic complications. However, additional research in animal models is required to clarify anti-glycation effects of IFA.
